# miRNA repertoire and host immune factor regulation upon avian coronavirus infection in eggs

**DOI:** 10.1007/s00705-020-04527-4

**Published:** 2020-02-06

**Authors:** Vera Kemp, Andrea Laconi, Giulio Cocciolo, Alinda J. Berends, Timo M. Breit, M. Hélène Verheije

**Affiliations:** 1 Faculty of Veterinary Medicine, Department Biomolecular Health Sciences, Division Pathology, Utrecht University, Utrecht, The Netherlands; 2 Department of Veterinary Medicine, University of Bari, Valenzano, Italy; 3 RNA Biology and Applied Bioinformatics Research Group, Swammerdam Institute for Life Sciences, Faculty of Science, University of Amsterdam, Amsterdam, The Netherlands; 4 Present Address: Department of Comparative Biomedicine and Food Science, University of Padua, Legnaro, Italy

## Abstract

Avian infectious bronchitis virus (IBV) is a coronavirus with great economic impact on the poultry industry, causing an acute and highly contagious disease in chickens that primarily affects the respiratory and reproductive systems. The cellular regulation of IBV pathogenesis and the host immune responses involved remain to be fully elucidated. MicroRNAs (miRNAs) have emerged as a class of crucial regulators of numerous cellular processes, including responses to viral infections. Here, we employed a high-throughput sequencing approach to analyze the miRNA composition of the spleen and the lungs of chicken embryos upon IBV infection. Compared to healthy chicken embryos, 13 and six miRNAs were upregulated in the spleen and the lungs, respectively, all predicted to influence viral transcription, cytokine production, and lymphocyte functioning. Subsequent downregulation of NFATC3, NFAT5, SPPL3, and TGFB2 genes in particular was observed only in the spleen, demonstrating the biological functionality of the miRNAs in this lymphoid organ. This is the first study that describes the modulation of miRNAs and the related host immune factors by IBV in chicken embryos. Our data provide novel insight into complex virus-host interactions and specifically highlight components that could affect the host’s immune response to IBV infection.

**Electronic supplementary material:**

The online version of this article (10.1007/s00705-020-04527-4) contains supplementary material, which is available to authorized users.

## Introduction

Avian infectious bronchitis virus (IBV) is an enveloped virus with a positive-stranded RNA genome. IBV is a member of the genus *Gammacoronavirus*, family *Coronaviridae* and is an important viral pathogen in the poultry industry. It causes a highly contagious disease in chickens, mainly affecting the respiratory and reproductive tract [1]. The mechanisms involved in IBV pathogenesis and the host’s response are still not fully understood. The pathogenicity of the virus has been linked to the activation of apoptosis, the inhibition of innate antiviral immunity, and the induction of proinflammatory cytokine production [2, 3]. However, the specific factors facilitating these processes, and the way IBV influences them for the benefit of either the virus or the host remain to be identified.

MicroRNAs (miRNAs) are small non-coding RNA molecules with a typical size of about 22 nucleotides (nt). They are implicated in numerous cellular processes, including viral infections. Several members of the family *Coronaviridae* have been shown to modulate the host miRNA repertoire, thereby influencing viral pathogenesis [4–9]. Coronavirus-induced miRNAs have been suggested to modulate the host’s antiviral immune responses including viral sensing [5], cytokine secretion [4, 7–9], and T-cell-mediated cell killing [7]. For IBV, the production of a number of miRNAs have been described to be influenced in chicken kidneys upon infection with IBV strains with different nephropathogenicity [10]. Little is known, however, about the overall effects of IBV infection on the miRNA repertoire influencing the (antiviral) immune response in particular.

In the present study, we examined the effect of the prototype IBV strain M41 on the miRNA composition in the spleen, a key organ in systemic immunity, and in the lungs of inoculated chicken embryos. We observed the upregulation of several miRNAs related to processes involved in viral replication and immune responses such as cytokine production and lymphocyte activities in both tissues. mRNA downregulation of the corresponding target genes was observed only in the spleen. Altogether, our findings provide novel insight into the cellular factors influenced by IBV infection, highlighting specific miRNAs involved in the host response to IBV.

## Materials and methods

### Eggs, viruses, and tissue collection

IBV strain M41 (Animal Health Service, Deventer, The Netherlands) was previously propagated and titrated in specific-pathogen-free (SPF) embryonated chicken eggs (ECEs). For the production of a virus stock, ten 8-day-old ECEs were inoculated with 100 times the 50% embryonic infectious dose (EID_50_), incubated for 48 hours (h), and subsequently cooled for 16–24 h prior to harvesting and pooling the allantoic fluid. Virus titration *in ovo* was based on the determination of the EID_50_ per ml, as determined at 7 days postinfection (dpi) according to the Reed and Muench method [11].

For infection experiments, eight fertilized SPF white leghorn eggs (Animal Health Service, Deventer, The Netherlands) were incubated at 37.5°C and 45–65% relative humidity. At fifteen days of age, the ECEs were inoculated via the allantoic cavity. Four ECEs were inoculated with 100 EID_50_ of M41 in 100 μl, and four with 100 μl of PBS control. The ECEs were candled twice daily. At the indicated time points after infection, two M41-infected and two PBS-inoculated ECEs were transferred to 4°C for 24 h prior to collection of the spleen and lungs. After collection, the tissues were immediately stored at -150°C until (mi)RNA isolation.

### miRNA isolation, cDNA library preparation and next-generation sequencing

miRNA isolation, cDNA library production and next-generation sequencing (NGS) were performed by the laboratories of Swammerdam Institute for Life Sciences at the University of Amsterdam. SmallRNA was isolated from the tissues using a mirVana™ miRNA Isolation Kit (Thermo Fisher Scientific), following the manufacturer protocol. Prior to cDNA library production, smallRNA purity and concentration were assessed for each sample using an Agilent RNA Screen Tape System© (Agilent Technologies, CA, USA). For each sample, a labelled cDNA library was produced using an Ion Total RNA-Seq Kit v2 for Small RNA Libraries (Thermo Fisher Scientific) according to the manufacturer’s instructions. The resulting sixteen labelled cDNA libraries, derived from the two tissues from eight embryos in total, were sequenced using an Ion PI™ Hi‑Q™ Sequencing 200 Kit (Thermo Fisher Scientific) for Ion Torrent technology, following the manufacturer’s protocol.

### Bioinformatics identification of miRNAs

Prior to analysis, reads containing poly-N, with 5’ adapter contaminants, without a 3’ adapter or the insert tag, containing poly-A or -T or -G or -C, and low-quality reads were removed from the dataset. After this first screening, sequences of 15-100 nt were defined as clean reads and were processed for further downstream analysis. The smallRNA reads were mapped against the *Gallus gallus* reference genome sequence [12] and the IBV M41 sequence (DQ834384.1). The mapped smallRNA reads were examined for the presence of known miRNAs using MiRBase20.0 (http://www.mirbase.org/).

### Differential expression analysis

To analyze the differential expression of the miRNAs, the number of reads for each sample was normalized to the total number of reads. The normalized data were used to calculate the mean of duplicate reads. The means of the normalized reads were used for downstream analysis. Differential expression analysis was performed within the same tissue at a specific time point, comparing uninfected and infected tissues at 48 and 72 hours postinfection (hpi). Fisher’s exact test was used to confirm differentially expressed (DE) miRNAs (*p*-value < 0.5) at a 5% false discovery rate (FDR) (Q value < 0.5) for the miRNAs showing counts > 100 reads and differential ≤ -0.6 or ≥ 0.6.

### Identification of target genes

Potential target genes of DE miRNAs were identified using the TargetScan 7.1 (http://www.targetscan.org) and miRBD (http://mirdb.org/) web platforms. Only targets identified by both programs were considered significant.

### Gene Ontology enrichment analysis

Gene Ontology enrichment analysis was carried out on the target gene candidates for the DE miRNAs. Biological processes with a fold enrichment > 2 and a *p*-value < 0.05 were considered significant.

### RNA isolation, and RT-qPCR analysis of target gene mRNA expression

RNA was isolated from 30 mg of tissue using an RNeasy Mini Kit (QIAGEN, catalog no. 74106). Prior to downstream RT-qPCR analysis, RNA concentrations were determined using a NanoDrop ND-1000 spectrophotometer (Isogen Life Science, de Meern, The Netherlands), and an additional DNase digestion was performed. Fivefold dilutions of the RNA samples were used for cDNA synthesis and subsequent RT-qPCR, using an iScript cDNA Synthesis Kit (Bio-Rad, catalog no. 170-8890) and an iTaq Universal SYBR Green One-Step Kit (Bio-Rad, catalog no. 172-5150). The thermal cycling protocol included the following steps: 10 min at 50°C (cDNA synthesis), 1 min at 95°C, 40 cycles of 10 s at 95°C and 30 s at 60°C, and a 60-90°C melt curve, with a 0.2°C increment every 5 seconds. The primers used (Biolegio, Nijmegen, The Netherlands) were NFAT3C_FW1 (CTCCTAGAACTAGCATTACAGATG), NFAT3C_RV1 (GACCAGGTGATGGAGTTGGAG), NFAT5_FW1 (CACTGAGGTGCCACGTAAATC), NFAT5_RV1 (GCTTTTGAGTTGCCTTTGCTG), SPPL3_FW2 (GTAGCAGACTATTACCTACGTG), SPPL3_RV2 (GAAGCTTCAGTTTGCCTAACTG), TGFB2_FW1 (GCAAGATTT GCAGGTATTGATGAC), TGFB2_RV1 (CCTGCACATTCCTAAAACAA), JUN_FW1 (GCAGAGCATGACGCTGAACCTG), JUN_RV1 (CTTGCTCGTCGGTAACGTTC), IBV5’GU391_fw (GCTTTTGAGCCTAGCGTT), IBV5’GL533_rv (GCCATGTTGTCACTGTCTATTG), housekeeping gene GAPDH_FW92 (GAAGGCTGGGGCTCATCTG), and GAPDH_RV92 (CAGTTGGTGGTGCACGATG), and housekeeping gene ACTB_FW89 (CAACACAGTGCTGTCTGGTGGTA), and ACTB_RV89 (ATCGTACTCCTGCTTGCTGATCC). All primers were validated for their sensitivity and specificity using fivefold dilutions of spleen and lung RNA samples obtained from 15-day-old chicken embryos. All samples were measured in duplicate. The expression levels of NFATC3, NFAT5, SPPL3, TGFB2, and JUN were normalized to the reference genes GAPDH and ACTB.

### Statistical analysis

Statistical analysis was performed using GraphPad Prism software (version 8.0.1). Data represent the mean and standard error of the mean. The means of the mock and IBV groups were compared using multiple Mann-Whitney tests. Significant differences are indicated by asterisks, with *p-*values < 0.05 shown as * and *p*-values < 0.01 as **. Non-significant differences are indicated by n.s.

## Results

### Identification of miRNAs

**Fig. 1:**
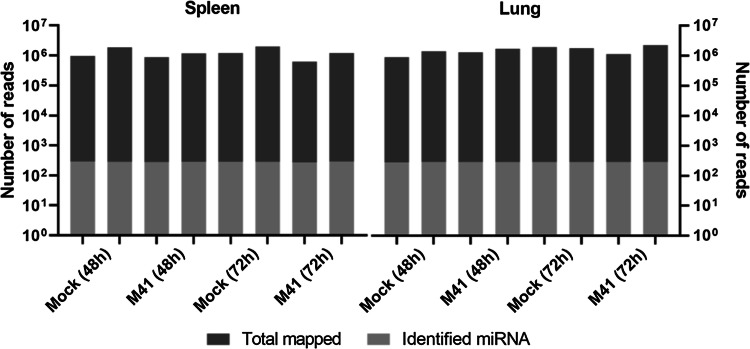
Number of smallRNA reads, mapped reads, and identified miRNAs. ECEs were inoculated with IBV or PBS, spleens and lungs were collected at 48 and 72 hpi, and RNA was isolated. High-throughput sequence analysis was performed, and the high-quality smallRNA sequences were mapped against the genome of *Gallus gallus*. The number of reads mapped against the *Gallus gallus* genome is shown in dark grey, and the number of conserved miRNAs identified is shown in light grey

To study the effect of IBV infection on the host repertoire of miRNAs that could influence the (antiviral) immune response, we analyzed the miRNA composition in the spleens and lungs of IBV-infected chicken embryos at two different time points. To this end, 15-day-old embryonated chicken eggs were inoculated with the prototype IBV strain M41. At 48 and 72 hpi, the tissues were collected and RNA was isolated. After high-throughput sequence analysis and selection of clean reads, the high-quality smallRNA sequences were mapped against the genome sequences of *Gallus gallus* and IBV M41. The number of reads matching the *Gallus gallus* genome is reported in Fig. 1. None of the smallRNA sequences detected in the infected tissues matched the IBV M41 genome.

To identify miRNAs, the detected smallRNA sequences were used in a BLAST search against known mature and immature miRNA sequences in the miRBase database. All reads with 1-4 nucleotide mismatches to a given miRNA sequence in miRBase were considered loose matches. Such mismatches might have arisen from sequencing errors, especially when occurring in counts with few reads, mutations and/or RNA editing events. The number of smallRNAs detected, the number of reads mapped to the *Gallus gallus* genome, and the number of miRNAs identified were similar among the different tissues and were not substantially influenced by IBV infection (Fig. 1). In total, between 266 and 288 different miRNAs were identified in the mock- and M41-infected tissues at 48 and 72 hpi.

### Differentially expressed miRNAs in M41- vs. mock-infected tissues

**Fig. 2:**
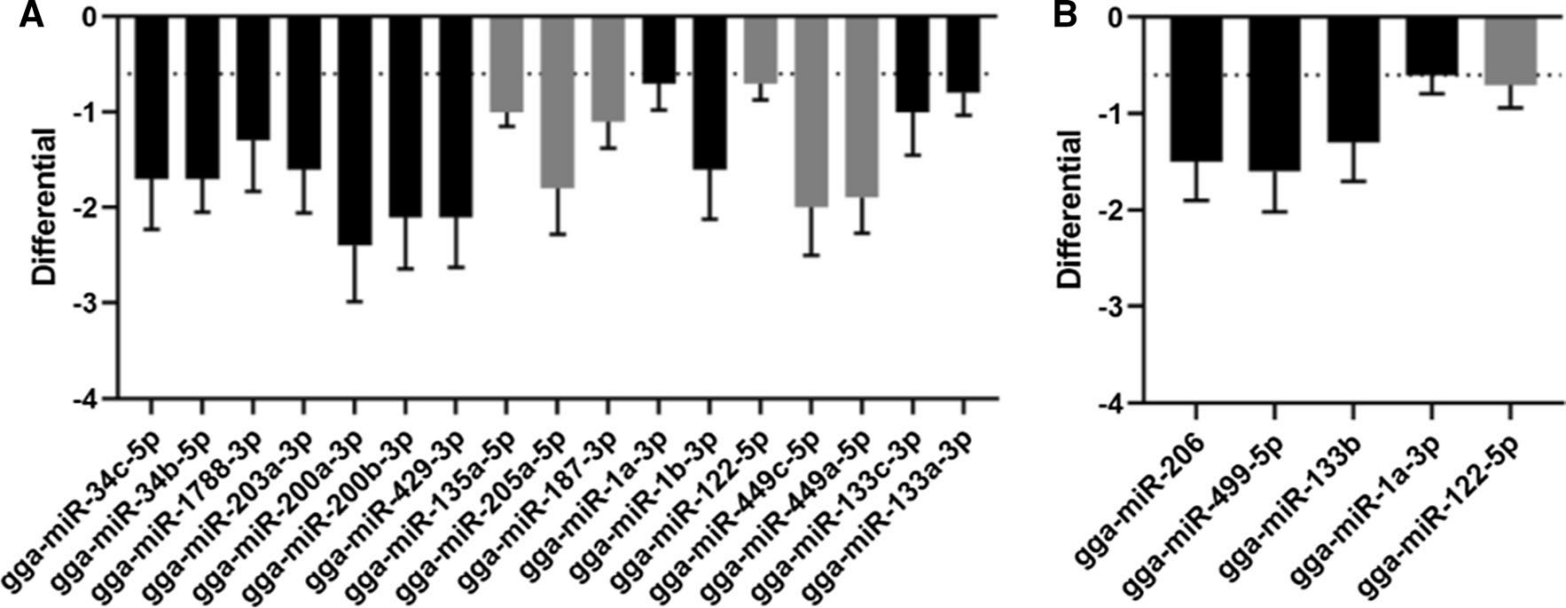
DE miRNAs in spleen and lungs at 72 hpi. Seventeen DE miRNAs were identified in the spleen **(A)**. Five DE miRNAs were identified in the lungs **(B)**. Grey bars represent miRNAs that were excluded from subsequent analysis because no common target gene could be identified. Dotted lines represent the cutoff value for differential expression (-0.6)

For both time points, the miRNA reads of the M41-infected tissues were compared with those of the mock-infected tissues. Based on our inclusion criteria (> 100 counts, differential > 0.6 or < -0.6, *p*-value < 0.05 and false discovery rate [FDR] < 0.05), no differentially expressed (DE) miRNAs were identified at 48 hpi in the spleen or in the lungs (data not shown). At 72 hpi, we identified seventeen DE miRNAs in the spleen (Fig. 2A) and seven DE miRNAs in the lung (Fig. 2B); all DE miRNAs were overexpressed in M41-infected tissues. An overview of the miRNAs prior to inclusion analysis based on our criteria can be found in the heat maps in Supplementary Fig. 1A (spleen) and B (lung).

### miRNAs target gene and Gene Ontology analysis

Potential targets of the DE miRNAs were identified using the TargetScan and miRBD online-based platforms. Only target genes identified using both platforms were considered trustworthy and are shown in Table 1. For seven miRNAs, no common target gene was identified by the two scan platforms. Therefore, these were left out of any further analysis. Gene Ontology (GO) analysis was performed to determine the biological processes in which the genes are involved, revealing that 13 immune-related GO terms were significantly enriched (*p* < 0.05) in the spleen, and six in the lungs. For the spleen, functional terms included positive regulation of B-cell development, induction of cytokine production, suppression of T-cell development, and positive regulation of viral transcription by the host. In the lungs, the functional terms were induction of cytokine production, B-cell proliferation, and regulation of the MAPK cascade.

**Table 1:**
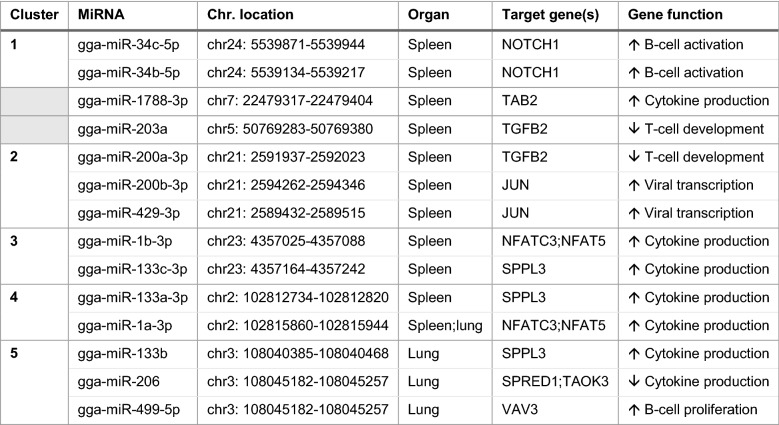
DE miRNAs in spleen and lung, the corresponding target gene, and gene functions

All DE miRNAs with identifiable gene targets are shown, including the predicted common gene functions. Based on genomic location, five miRNA clusters were identified. Two identified miRNAs were not located in clusters (indicated by grey colour)

### Clustering of miRNAs

For each DE miRNA, its genomic locus was identified using miRBase (Table 1). The analysis revealed that some of the DE miRNAs were located on the same chromosome and in close proximity to each other. Accordingly to previous studies, miRNAs can be considered part of the same cluster if they are less than 10 kb apart [13]. Considering this, nine of the DE miRNAs in the spleen at 72 hpi could be assigned to four clusters; each of them contained two to three miRNAs. In the lungs, the analysis revealed that three DE miRNAs belonged to one cluster.

### Gene expression levels of selected target genes

**Fig. 3:**
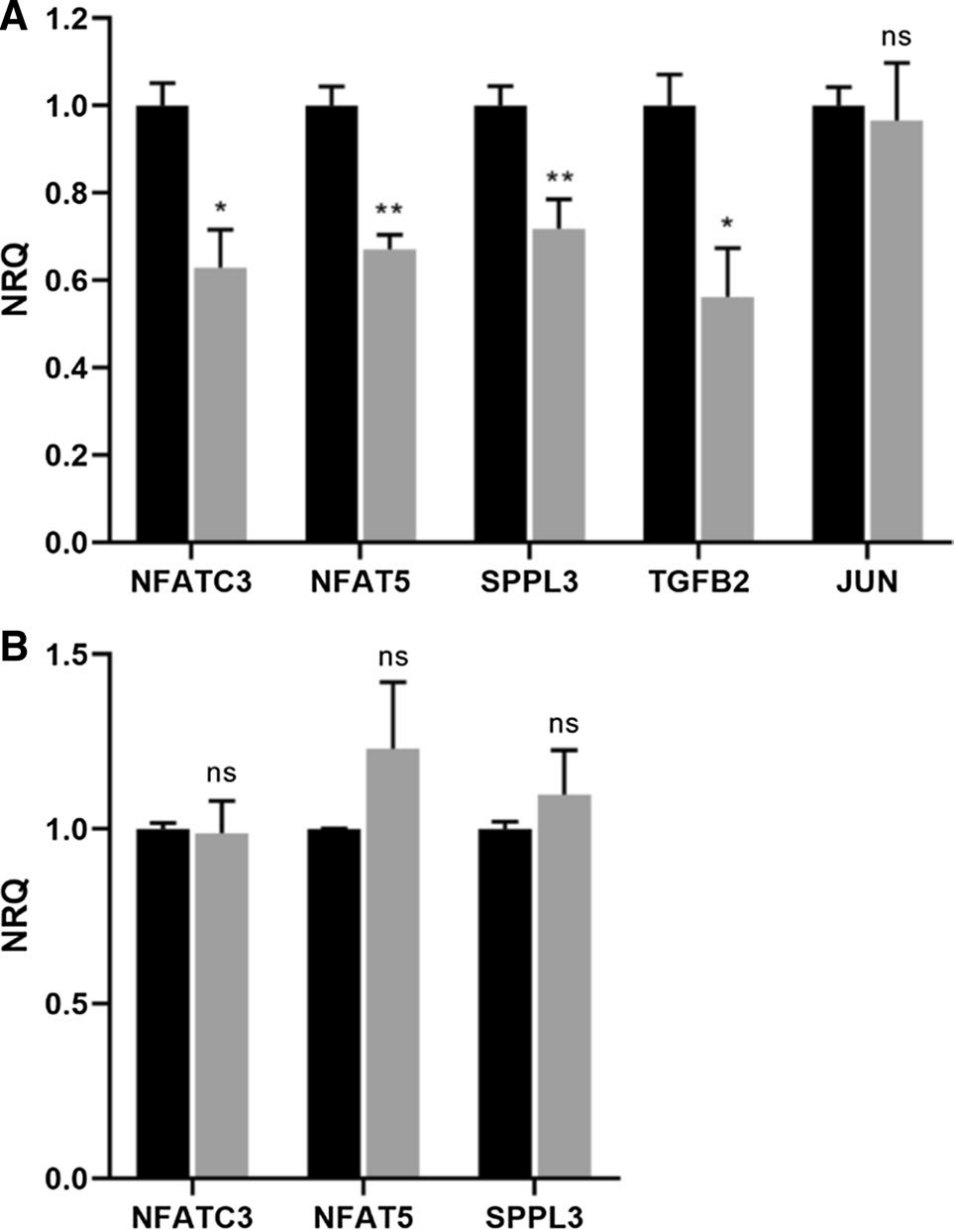
Relative gene expression of selected target genes in spleen and lungs. ECEs were inoculated with IBV (grey bars) or PBS (black bars). At 72 hpi, embryo organs were collected and RNA was isolated, followed by RT-qPCR analysis. The mean expression values, normalized for GAPDH and ACTB expression and relative to the PBS group are shown. Error bars represent the standard error of the mean. Significant differences between the means of the mock and IBV groups are indicated by asterisks, with *p*-values < 0.05 shown as * and *p*-values < 0.01 as **. Non-significant differences are indicated by ns. **A.** NFATC3, NFAT5, SPPL3, TGFB2, and JUN expression in the spleen. **B.** NFATC3, NFAT5, and SPPL3 expression in the lungs

To see whether the mRNA expression levels of the identified miRNA target genes are downregulated upon IBV infection, RT-qPCR analysis was performed on the spleens and lungs of M41-infected and mock-infected chicken embryos at 72 hpi. Successful IBV infection was confirmed in all M41-infected embryos by measuring viral RNA in the lungs (Cq ≤ 30). We selected three gene targets of DE miRNAs both in the spleens and in the lungs for which downregulation is predicted to negatively affect immune activities (NFATC3, NFAT5 and SPPL3). Moreover, we included two genes targeted by DE miRNAs only in the spleen (TGFB2 and JUN) for which downregulation would presumably result in immune activation. Figure 3 shows the normalized relative expression of the genes, demonstrating gene downregulation in the spleens (Fig. 3A) upon M41 infection for all genes except JUN. The RT-qPCR analysis of the lungs revealed that M41 infection does not influence the expression of any of the analyzed genes in this organ (Fig. 3B).

## Discussion

In this study, we analyzed the miRNA repertoire of chicken embryos upon infection with IBV. In particular, we identified miRNAs with possible functions in antiviral immunity that were functional in downregulation of target gene mRNAs in the spleen, but not in the lungs of embryos. Twelve of the 14 identified DE miRNAs with a common predicted target gene clustered in four specific chromosomal regions as pairs or groups of three, suggesting a shared mechanism for their deregulation and strengthening the likelihood of their involvement in IBV infection.

Notably, four of the five predicted target genes that were analyzed were downregulated in the spleen, indicating that the identified miRNAs are functional. In contrast, in the lungs, none of the predicted target genes were affected, despite the presence of viral RNA. Theoretically, immune-related genes should be affected to a larger extent in organs with an important immune function. Furthermore, the number of affected miRNAs and miRNA clusters was higher in the spleen than in the lungs (17 versus five DE miRNAs, and four versus one cluster), suggesting that widespread host miRNA modulation occurs in the spleen. However, an effect in the lungs at a different time point cannot be ruled out. As primary immune responses against virus infections often take days to fully develop, especially adaptive antiviral immune responses including lymphocyte expansion and activation, it could be worthwhile to examine the modulation of immune-related target genes at later time points. Finally, mRNA targets might be translationally repressed in the lungs without inducing degradation, but this was not further investigated.

All of the identified miRNAs with a common predicted target gene have been described before to be modulated in viral or bacterial infections. An extensive overview of the literature on this is shown in Table 2. The majority of the miRNAs have been associated with virus replication and/or host immune responses. Interestingly, the vast majority of these miRNAs had been found previously in association with various bacterial or viral infections of chickens in particular, which is suggestive of a common host response of chickens to pathogenic infections. A wide range of miRNAs, including miR-34c, -34b, -1b, -1a, -206, and -499, have been linked to influenza virus infection in chickens, and most of these miRNAs have been associated with immune-related functions (miR-34c, -34b, -1b, -1a, and -206). As influenza viruses and coronaviruses share some of their immune-modulating activities [14], the chicken miRNAs that have been identified might have important and similar roles in immune modulation in infections with different (RNA) viruses.

**Table 2:** Descriptions of miRNAs described in the literature

miRNA	Species	Cell type/tissue	Infection	Findings
MiR-34c	Human	CD4^+^ T-cells	HIV	T-cell activation, facilitates virus replication [15]
	Human	HeLa	Flaviviruses	Inhibits virus replication, Wnt/Notch/IFN-mediated [16]
	Chicken	Trachea	Influenza A virus	Upregulated upon H5N3 infection [17]
	Human	A549	Influenza A virus	Enhances virus replication [18]
MiR-34b	Chicken	Spleen	ALV	Promotes virus replication by targeting MDA5 [5]
	Human	Throat swab	Influenza B virus	Upregulated upon Influenza B virus infection [19]
	Chicken	Spleen	Marek’s disease	Upregulated in virus-resistant line [20]
	Human	Huh7.5	HCV	Facilitates virus replication [21]
	Chicken	Trachea	Influenza A virus	Upregulated upon H5N3 infection [17]
MiR-1788	Chicken	Cecum	Salmonella	Upregulated upon S. Typhimurium infection [22]
MiR-203a	Chicken	DF-1 & embryo	NDV	Enhances virus replication and embryonic death [23]
	Pig	LFBK-avβ6	FMDV	Inhibits FMDV replication [24]
	Human	HEPG2	HBV	Upregulated upon infection, induces inflammation [25]
	Human	HEPG2	HCV	Downregulated → EMT & carcinogenesis [26]
	Human	Nasal mucosa	RSV	Upregulated in RSV-positive infants [27]
MiR-200a	Human	HEPG2	HBV	Downregulated upon infection → cell division & invasion [28]
	Chicken	Intestine	Marek’s disease	Upregulated in virus-susceptible line [20]
MiR-200b	Human	CNE1^AKATA^	EBV	Induces EBV lytic reactivation [29]
	Chicken	Intestine	Marek’s disease	Upregulated in virus-susceptible line [20]
MiR-429	Human	EBV-293	EBV	Induces EBV lytic reactivation [29]
	Human	Nasal mucosa	RSV	Downregulated in RSV-positive infants [27]
	Chicken	Intestine	Marek’s disease	Upregulated in virus-susceptible line [20]
MiR-1b	Chicken	Trachea & lung	Influenza A virus	Upregulated upon H5N3 infection [17]
MiR-133c	Chicken	Cecum	Salmonella	Upregulated upon *S. enterica* infection [30]
MiR-133a	Monkey	Vero	DENV	Suppresses viral replication [31]
MiR-1a	Chicken	Kidney	IBV	Upregulated upon viral infection [10]
	Chicken	Trachea & lung	Influenza A virus	Upregulated upon H5N3 infection [17]
MiR-133b	Dog	Lung	Influenza A virus	May inhibit innate immunity, increasing pathogenicity [32]
MiR-206	Chicken	Lung	Influenza A virus	Downregulated upon infection [33]
	Pig	Lung	Influenza A virus	Upregulated upon H1N2 infection [34]
	Chicken	Trachea & lung	Influenza A virus	Upregulated in lung; downregulated in trachea (upon H5N3 infection) [17]
MiR-499	Chicken	Spleen	Marek’s disease	Upregulated in virus-resistant line [20]
	Chicken	Trachea	Influenza A virus	Upregulated upon H5N3 infection [17]

HIV, human immunodeficiency virus; ALV, avian leukosis virus; HCV; hepatitis C virus; NDV, Newcastle disease virus; FMDV, foot and mouth disease virus; HBV, hepatitis B virus; RSV, respiratory syncytial virus; DENV, dengue virus; IBV, infectious bronchitis virus; HeLa, cervical cancer cell line; A549, human lung epithelial cell line; Huh7.5, human hepatocellular carcinoma cell line; DF-1, chicken fibroblast cell line; LFBK-avβ6, fetal porcine kidney cell line; HEPG2, human hepatocellular carcinoma cell line; CNE1^AKATA^, CNE1 cell line with latent EBV infection; EBV-293, 293 cell line with latent EBV infection; MDA5, melanoma differentiation-associated protein 5; EMT, epithelial-mesenchymal transition

A previous study revealed DE miRNAs in chicken kidneys upon infection with IBV strains with different virulence [10]. Ten of the identified miRNA hits in that study were also DE in our study, including gga−miR−206, −499, −133b, −1a, −133c, and −133a, all of which have been reported to be involved in immune functions such as cytokine production. Perhaps these miRNAs represent a general chicken host response to infection with virulent IBV. Notably, some upregulated miRNAs in our screening were found to be downregulated in their screening, namely gga−miR−203a, −200a, and −429. These miRNAs may have opposing roles in virus-host interactions depending on the viral strain or organ. Interestingly, miRNAs such as gga-miR-203a and -200a target genes with suspected pro-viral functions, such as TGFB2, a gene involved in T-cell suppression, which might result in stimulation of antiviral T-cell functions. Gga-miR-429-3p may inhibit viral replication by targeting JUN, which normally stimulates viral transcription by the host. Taken together, the upregulation of these miRNAs upon M41 infection could represent an antiviral host response.

Our study provides novel insight into the complex interaction between IBV and the chicken host, and specifically the factors that could influence the immune response to IBV infection. These results will contribute to our understanding of the mechanisms involved in IBV infection and pathogenesis.

## Electronic supplementary material

Below is the link to the electronic supplementary material.

Supplementary material 1 (TIFF 1007 kb)

Supplementary material 2 (TIFF 989 kb)

## Abbreviations

ACTB: beta-actin
Cq: cycle quantification
DE: differentially expressed
dpi: days postinfection
ECEs: embryonated chicken eggs
EID_50_: 50% embryonic infectious dose
FDR: false discovery rate
GAPDH: glyceraldehyde-3-phosphate dehydrogenase
GO: Gene Ontology
hpi: hours postinfection
h: hours
IBV: infectious bronchitis virus
JUN: Jun proto-oncogene
AP-1: transcription factor subunit
Kb: kilobase
MAPK: mitogen-activated protein kinase
miRNA: microRNA
NFAT5: nuclear factor of activated T-cells 5
NFATC3: nuclear factor of activated T-cells, cytoplasmic 3
NGS: next-generation sequencing
n.s.: non-significant
qRT-PCR: quantitative real-time polymerase chain reaction
SPPL3: signal peptide peptidase-like 3
TGFB2: transforming growth factor beta-2
SPF: specific-pathogen-free
