# Supplementary figures and images for: miRNA repertoire and host immune factor regulation upon avian coronavirus infection in eggs
Source: Arch Virol. 2020 Feb 6;165(4):835–43. doi: 10.1007/s00705-020-04527-4 (PMC7086581; doi:10.1007/s00705-020-04527-4)

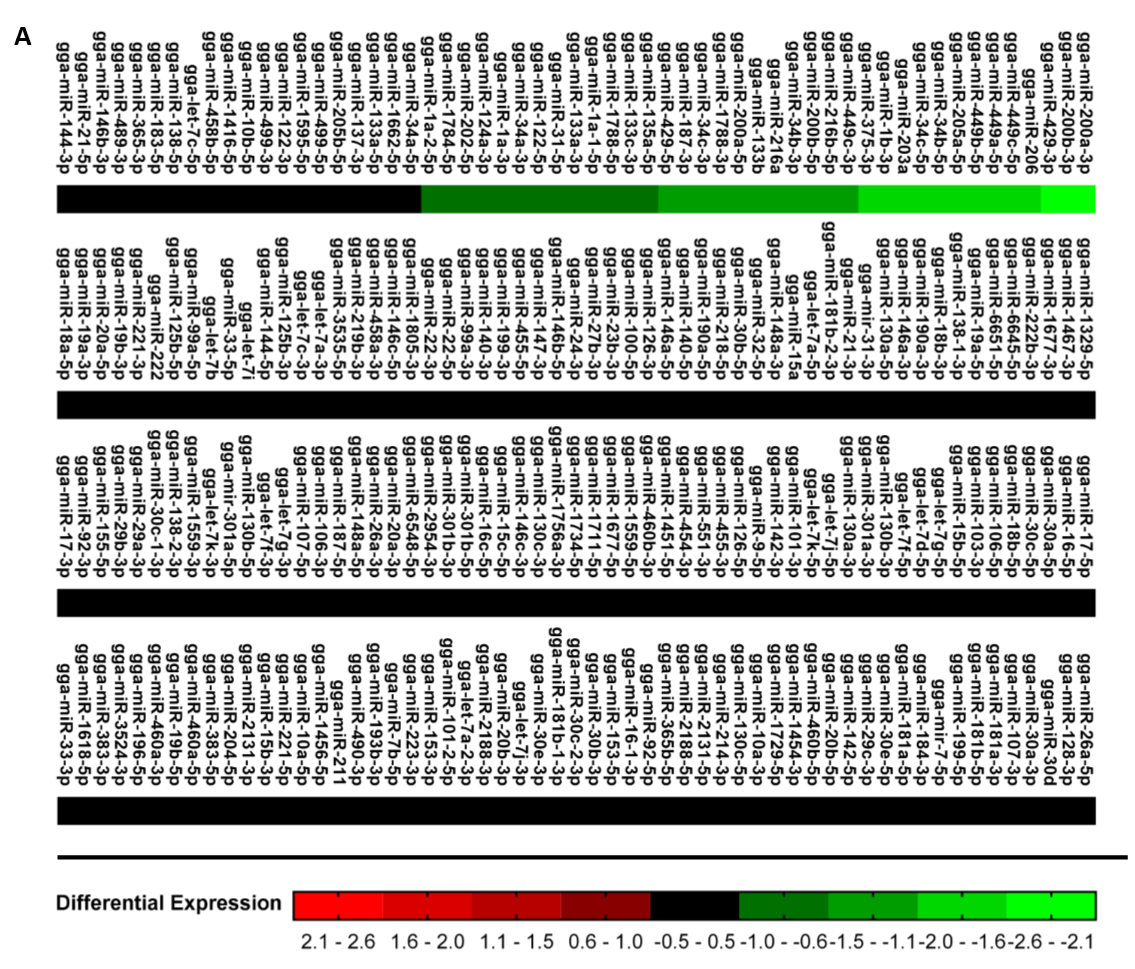

Supplement: Supplementary file 1 — Supplementary material 1 (TIFF 1007 kb) [file 705_2020_4527_MOESM1_ESM.tif]

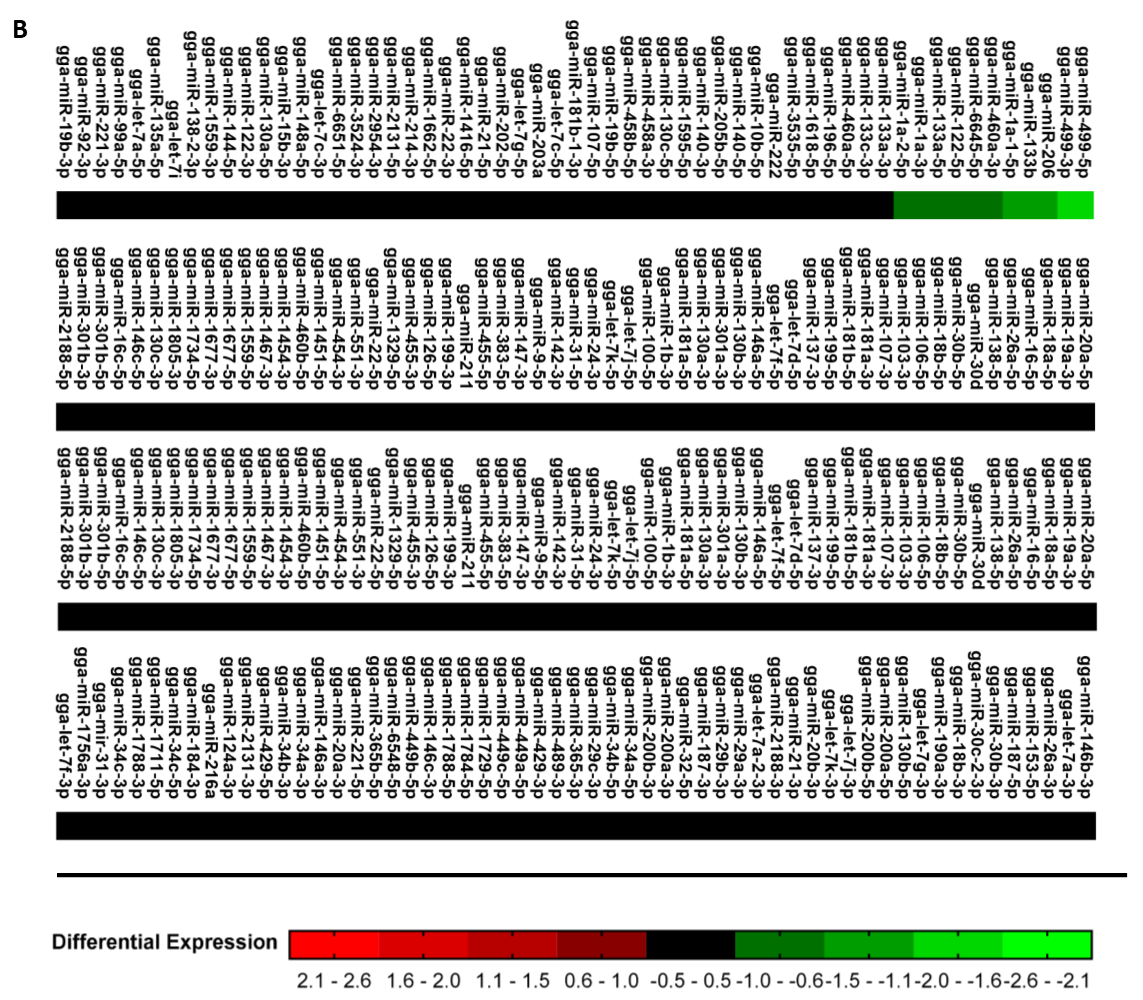

Supplement: Supplementary file 2 — Supplementary material 2 (TIFF 989 kb) [file 705_2020_4527_MOESM2_ESM.tif]
